# Substrate-Free InGaN/GaN Nanowire Light-Emitting Diodes

**DOI:** 10.1186/s11671-015-1143-5

**Published:** 2015-11-17

**Authors:** Vladimir Neplokh, Agnes Messanvi, Hezhi Zhang, Francois H. Julien, Andrey Babichev, Joel Eymery, Christophe Durand, Maria Tchernycheva

**Affiliations:** Institut d’Electronique Fondamentale, UMR CNRS 8622, University Paris Sud 11, 91405 Orsay, France; Univ. Grenoble Alpes, 38000 Grenoble, France; CEA, INAC-SP2M, “Nanophysique et semiconducteurs” group, 38000 Grenoble, France; ITMO University, 197101 Saint Petersburg, Russia; Ioffe Institute, Polytechnicheskaya 26, 194021 St. Petersburg, Russia

**Keywords:** Nanowire, Light-emitting diodes, Substrate-free devices Gallium nitride, 85.30.-z, 85.60.-q, 82.35.-x, 85.35.Be, 81.07.Gf, 81.07.St, 85.60.Bt, 85.60.Dw, 85.60.Jb, 82.35.Gh, 81.07.Pr

## Abstract

We report on the demonstration of substrate-free nanowire/polydimethylsiloxane (PDMS) membrane light-emitting diodes (LEDs). Metal-organic vapour-phase epitaxy (MOVPE)-grown InGaN/GaN core–shell nanowires were encapsulated into PDMS layer. After metal deposition to p-GaN, a thick PDMS cap layer was spin-coated and the membrane was manually peeled from the sapphire substrate, flipped upside down onto a steel holder, and transparent indium tin oxide (ITO) contact to n-GaN was deposited. The fabricated LEDs demonstrate rectifying diode characteristics. For the electroluminescence (EL) measurements, the samples were manually bonded using silver paint. The EL spectra measured at different applied voltages demonstrate a blue shift with the current increase. This shift is explained by the current injection into the InGaN areas of the active region with different average indium content.

## Background

Nitride light-emitting diodes (LEDs) have found numerous applications in our everyday life (general lighting, automotive headlamps, traffic signals, indicator lamps for electronic devices, etc.). Today, the market is dominated by two-dimensional devices based on thin film technology. To further boost the LED performance and to add new functionalities, three-dimensional nanomaterials have recently emerged [1, 2]. In particular, nitride high-aspect-ratio nanocrystals (referred to as nanowires (NWs)) allow to improve the material quality of the LED active region and to facilitate the light extraction [3]. The NW LED core–shell structure geometry also allows growth on non-polar *m*-planes to avoid undesirable quantum-confined Stark effect (QCSE).

The attractive feature of the NWs is that they provide high tolerance for the growth on lattice-mismatched substrates (e.g. growth of high-quality nitride NWs on silicon [4, 5] or sapphire). The concept of the ‘NW substrate independence’ can be further extended by transferring the NWs to other substrates (including non-crystalline materials) in either planar [6–9] or vertical [10–13] architectures. In this way, the NWs are used independently from their growth substrate, which can potentially be recycled. New functionalities for LEDs can be imagined. For example, NW LEDs can be mounted on metallic layers for efficient heat sinking or LEDs of different colours can be integrated on the same holder. In particular, flexible NW devices can be fabricated using this approach [12].

The first demonstration of nanowire flexible LEDs has been done using ZnO nanowires grown in solution on plastic substrate [14] demonstrating the electroluminescence in the visible range. However, the direct growth on the plastic substrate imposes to use a low temperature and strongly limits the range of accessible materials and growth techniques. To achieve high-efficiency visible emission, it is advantageous to make use of InGaN NWs elaborated at high temperature following standard epitaxial methods. The NWs can then be reported to a different substrate for LED fabrication. Recently, the first substrate-free nitride NW LED has been demonstrated employing the NW transfer [15]. The InGaN/GaN NWs were grown on a non-conventional Si/SiO_2_/graphene template, embedded into a polymer and separated from their growth substrate by wet etching of a sacrificial SiO_2_ layer [15]. In this work, we propose a different fabrication approach based on a mechanical peel-off of the NWs with the method described in the literature [10, 16, 17], which does not require any sacrificial layer and thus facilitates the fabrication. Self-assembled core/shell NWs containing seven InGaN/GaN quantum wells (QWs) were grown by metal-organic vapour-phase epitaxy (MOVPE) on *c*-sapphire substrates. The NW structure was probed by cathodoluminescence evidencing two spectrally shifted emissions originating from the InGaN/GaN QWs located on the top polar minus *c*-plane and on the lateral non-polar *m*-plane facets. For the LED fabrication, the NWs were encapsulated into a polydimethylsiloxane (PDMS) layer and mechanically peeled from their growth substrate. The obtained NW/PDMS composite membrane was electrically contacted and fixed on a steel holder. The electrical characteristics of membrane NW LEDs presented a rectifying behaviour. The electroluminescence (EL) appeared starting from 5 V forward bias. The EL spectra exhibited two peaks at 412 and 466 nm, and the relative peak intensity changed with applied bias. In agreement with the cathodoluminescence mapping, this spectral behaviour is attributed to the current injection into the areas of the active InGaN/GaN QWs with different average In content located on the lateral and on the top NW facets.

## Methods

### Nanowire Growth and Device Fabrication

**Fig 1:**
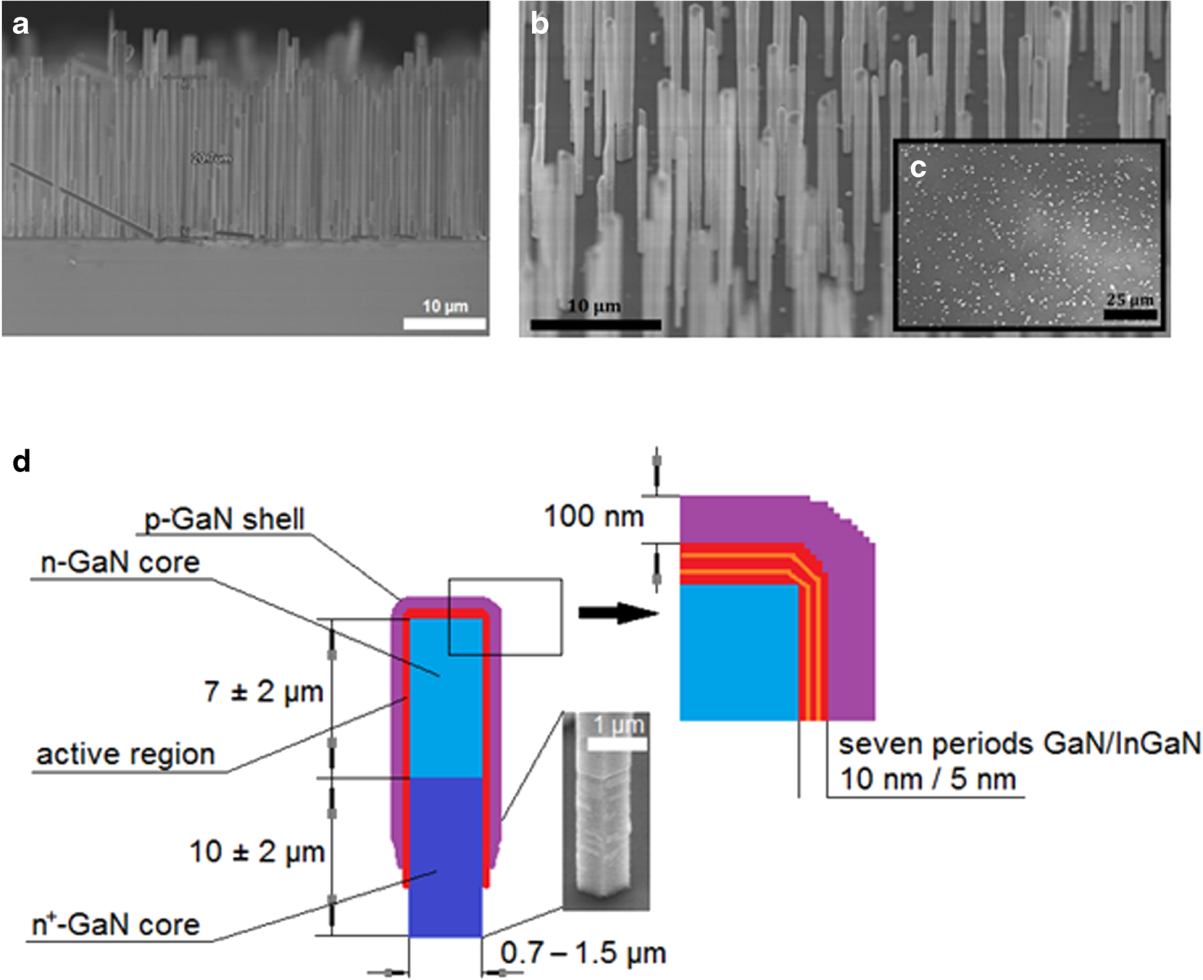
The GaN NW core–shell structure. **a** Cross-sectional SEM image of the as-grown NWs with the average height of ~20 μm. **b** 45° tilted SEM image of the NW array. **c** Top view SEM image of the NWs. **d** Schematic of the nanowire internal structure

The GaN core–shell NWs were grown on 2-in. sapphire substrates by self-assembled MOVPE, see details in [18, 19]. The growth started with 10 ± 2-μm-long n-doped GaN NWs with a diameter in the range 700–1500 nm (~10^20^ cm^−3^ concentration of Si doping atoms). We note that a SiN_*x*_ ultrathin layer is spontaneously formed around this wire part due to the high silane flux passivating the wire surface. Then, another 7 ± 2-μm GaN segment was grown without the silane flux, and the material is unintentionally doped by ~10^18^ cm^−3^ Si [20]. The active region (AR) was deposited directly on the NW surface by switching the growth conditions from axial growth to radial growth [19, 21]. Due to the presence of the SiN_*x*_ layer around the wire base, the radial growth is inhibited in the lower wire part and the core/shell heterostructure is only formed around the upper non-intentionally doped wire part, as described in [21]. The AR consisted of seven periods of 5-nm InGaN quantum wells delimited by 10-nm-thick GaN barriers with an indium content in the QWs about 15 % [19]. After growth of the AR, the p-GaN 100-nm shell layer was deposited, the hole concentration is estimated to be in the 10^16^–10^17^ cm^−3^ range [20]. The NW density is about 5 × 10^6^ per cm^−2^. The NW morphology and the internal structure are illustrated in Fig. 1a–d.

**Fig. 2:**
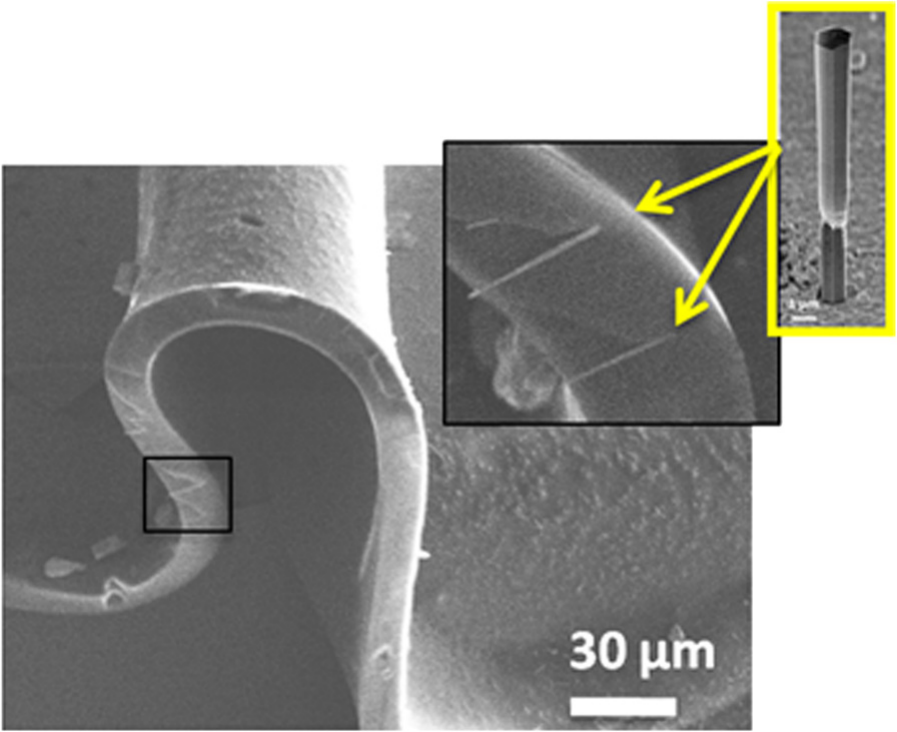
SEM image of the nanowire/PDMS membrane. *Inset* shows a high magnification SEM image of the region marked with a *black rectangle*, where single NWs are visible. The NWs are marked with *arrows*

For the fabrication of substrate-free NW LEDs, the nanowire embedding and lift-off procedure was first optimized on a series of test samples with a similar NW density. The samples were spin-coated with PDMS polymer with a 9:1 (base:cure agent) ratio to bury the NWs. The PDMS was cured at 80 °C for 50 min, and then the PDMS/NW composite layer was mechanically peeled off following the procedure described in the literature [10–12]. The PDMS/NW membrane was deposited on a metallic holder and imaged in a scanning electron microscope (SEM). Figure 2 shows an SEM image of the edge of the membrane, where several NWs can be distinguished. The layer is highly flexible. Despite the small curvature radius (~70 μm) of the region imaged in Fig. 2, the embedded NWs preserve their integrity, validating the possibility to manipulate and process the composite membranes for substrate-free device fabrication.

**Fig. 3:**
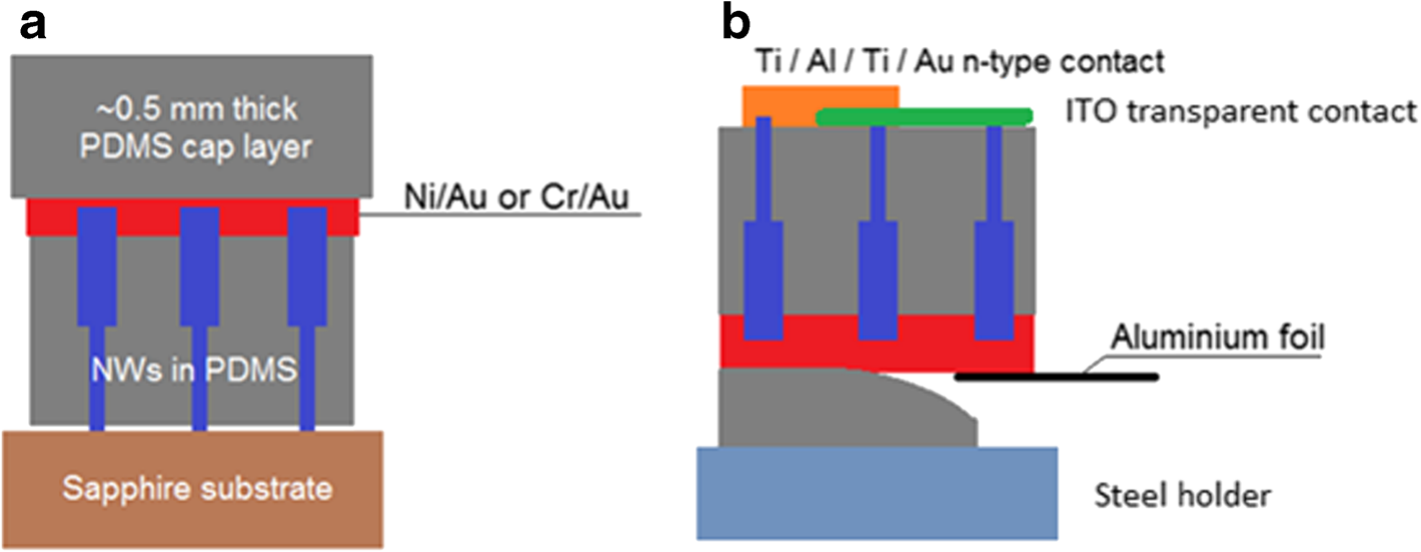
Schematic of the processing steps. **a** Encapsulated NWs with a metal contact to p-doped GaN shells and a PDMS cap layer before peeling. **b** Final device consisting of a flipped membrane with a transparent ITO contact to n-doped GaN NW base parts and top metal contact pads. The PDMS cap is partially released, and aluminium foil is attached to the bottom metal contact

For LED fabrication, the samples were spin-coated and cured to form a 18 ± 2-μm PDMS layer. To delete undesirable traces of PDMS on the tops of the NWs, the samples were etched in the CHF_3_/O_2_ plasma for 3 min. Then, they were treated with oxygen plasma for 5 min to modify the PDMS surface state in order to improve the adhesion of the metal contact [22]. The contact to the p-type GaN shell was made by depositing either 10-nm Ni/200-nm Au [23] or 10-nm Cr/200-nm Au [24]. Both Ni/Au and Cr/Au metallizations have resulted in similar performance; in the following, we describe the characterization of the device with Ni/Au contact. The fabrication process is schematically presented in Fig. 3a.

Thin free-standing composite membranes are rather difficult to manipulate since they are fragile and can easily roll up. Therefore, an additional PDMS cap layer (~0.5 mm thick, cured at 80 °C for 2 h) was deposited on the metal contact to be used as a mechanical support. It helps to peel off the structure from the sapphire substrate and prevents the peeled layer from rolling. After the mechanical lift-off of the nanowire/PDMS membrane, the sample was flipped upside down and put onto a steel holder for further processing and easy manipulation. Then, the second contact was deposited on the n-type GaN core bases of the NWs, which after the membrane flipping were located on its top part. The 100-nm-thick indium tin oxide (ITO) layer was deposited by sputtering through a shadow mask with an array of 0.5-mm-diameter circular openings [25]. After that, the shadow mask was slightly shifted and the 5-nm Ti/15-nm Al/5-nm Ti/100-nm Au metal pads were deposited to achieve a partial overlap between the metal and ITO (Fig. 3b); the corresponding work function suits ohmic injection in the n-GaN [26]. Thus, we obtain an array of LEDs, each consisting of a large number of parallel-connected NWs. The LEDs share the same bottom contact but can be addressed independently by their top metal pad.

To get access to the metal contact to p-GaN shells, which is between the 18-μm PDMS encapsulating layer and the 0.5-mm cap layer, the cap layer was mechanically released over a small area at the sample edge and a piece of aluminium foil was attached to the metallization with silver paint, as schematized in Fig. 3b. The ITO/metal contact to n-GaN NW base parts, which is accessible on the sample surface, was connected using metallic probes for the electrical characterizations. For EL spectroscopy, copper wires were manually attached to the metal pads using silver paint and a sharpened wooden toothpick since the composite NW/PDMS structure cannot stand the micro-bonding procedure.

## Results and Discussion

**Fig. 4:**
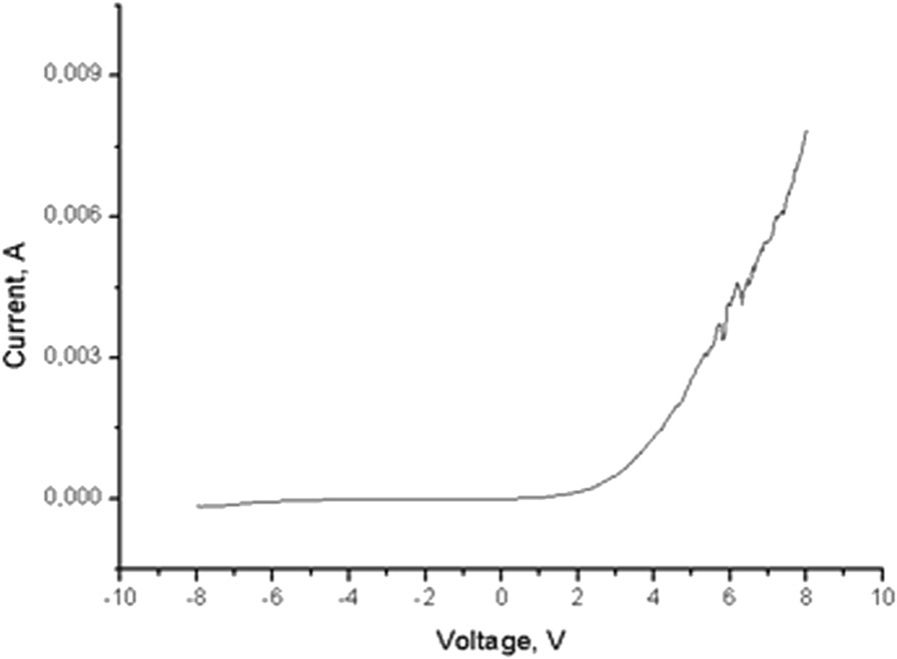
*I*–*V* curve of the nanowire/PDMS membrane LED

The fabricated membrane LEDs were electrically characterized in a Janis probe station coupled to a Keithley 2636 source meter. The electrical potential was applied to the bottom metal contact connected to the p-doped GaN shells, while the top transparent contact (ITO through Ti/Al/Ti/Au) was grounded. Figure 4 displays a current–voltage (*I*–*V*) characteristic at room temperature for a representative top contact pad. As expected from the NW structure, the *I*–*V* curve has a diode shape. The reverse leakage current is ~ 0.1 mA at −8 V compared to the direct current of ~10 mA at 8 V. As seen from Fig. 4, the *I*–*V* curves exhibit low-magnitude current instabilities for direct voltages higher than 5 V. These random current fluctuations are accompanied by LED emission blinking. The origin of this blinking is not completely understood. We suspect that these fluctuations may arise from the top ITO contact instability induced by Joule heating and underlying PDMS deformations. Alternative transparent contacts will be further optimized to achieve a stable emission.

**Fig. 5:**
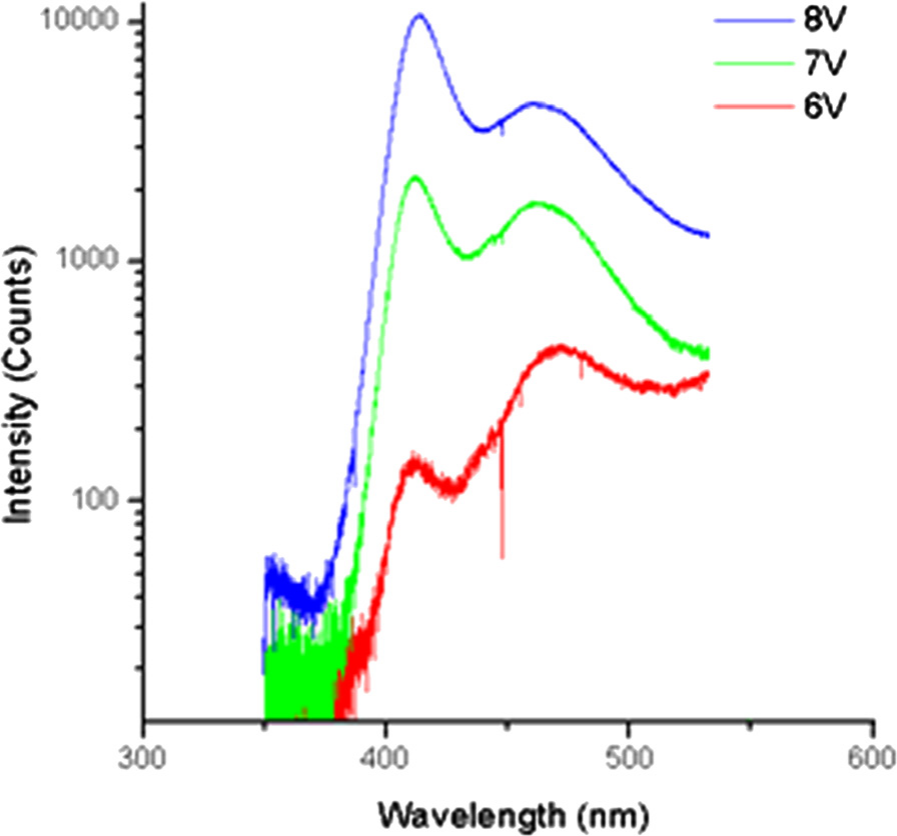
EL spectra at 6, 7, and 8 V

**Fig. 6:**
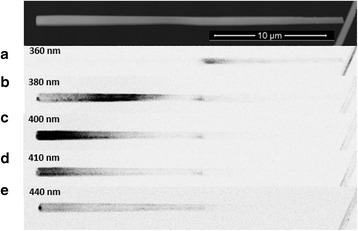
Filtered CL maps of a representative nanowire (**a**–**e**)

The EL spectra of the NW/PDMS membrane LEDs were measured at room temperature using the HR460 spectrometer equipped with a CCD camera. The EL spectra under different applied biases are presented in Fig. 5. The EL spectra exhibit two distinct peaks at 412 nm with a full width at half maximum (FWHM) of 20 nm and at 466 nm with a FWHM of 35 nm. For better understanding the origin of these peaks, cathodoluminescence (CL) mappings have been performed at 5 K on dispersed single wires (electron beam acceleration voltage = 20 kV and current = 1 nA, respectively). CL maps filtered for different detection wavelengths are displayed in Fig. 6(a–e). As it is seen in Fig. 6(b–d), the short-wavelength peak (380–410 nm) is associated with the emission of the radial QWs, whereas the long-wavelength peak (440 nm—Fig. 6(e)) arises from the axial QWs. Therefore, the two peaks observed in the EL spectra are attributed to the radial and axial QWs emission, respectively, as explained in detail in [27]. Based on the CL peak wavelengths and the confinement modelling with Silvaco software, we roughly estimate the average indium content *x* in the radial (*m*-plane) and axial (minus *c*-plane) In_*x*_Ga_1 − *x*_N QWs to be close to 10 ± 3 and 16 ± 4.5 %, respectively [28]. We conclude that the polar NW facet is more favourable for In incorporation than the radial facet in agreement with previous reports [29, 30].

The LED starts to luminesce at a forward bias of ~5 V with a working current in the order of 1 mA. This relatively high light-up voltage is attributed to the non-ohmic nature of the ITO contact to the moderately doped p-GaN shell. As previously reported in [27] for single NW LEDs, at low injection. the low energy peak is dominant. With increasing voltage, the short-wavelength peak increases faster than the long-wavelength peak. The short-wavelength peak becomes dominant at high injection (8 V, 10 mA). This relative intensity variation between the two peaks can be understood in terms of the redistribution of the injection current in the nanowire. The saturation current through the active region is exponentially dependent on the In content, and, for the mentioned difference in In content by several per cent, the saturation currents of the axial and radial active region parts are strongly different [31]. The high saturation current in the In-rich part favours the preferential injection in this region. However, as the injection increases, the current spreading in the resistive p-GaN shell part starts to play an important role [27]. The current is redistributed due to the potential drop in the shell, and the injection in the radial part of the active region, which in addition has a larger surface compared to the axial region, becomes favourable. It should also be noted that the radial QWs on the *m*-plane are expected to have less defects in comparison to the axial QWs due to their lower In content. This would also lead to the lower EL droop at higher currents for the *m*-plane QWs and therefore to a domination of the short-wavelength EL peak at high injection.

The increase of the injection current above 10 mA resulted in the LED failure. We believe that the device degradation is not caused by the NW structural degradation, but by the top contact failure, for which we observe a morphology change. This failure at a relatively low current can be attributed to the mechanical deformations of the soft PDMS layer and to the Joule heating. The degradation also can be caused by ion diffusion into polymer layer during current spreading in the ITO contact [32]. In the future contact optimizations, this effect can be suppressed by creating a thin metal layer between ITO and PDMS layers [33]. Alternative transparent contacts based on CVD-grown graphene layers can also be used [34].

The LED fabrication has been performed on several membranes showing good process reproducibility. EL measurements of the LED after 2-week storage in ambient air have not evidenced any device degradation.

## Conclusions

Substrate-free NW LEDs have been fabricated using the polymer embedding and peel-off procedure. The devices show rectifying electrical behaviour and emission in the blue spectral range. Further improvement of the transparent contact would make these LEDs competitive with the NW LEDs fabricated on rigid crystalline substrates. The developed fabrication approach allows for sapphire substrate recycling, which is important for the LED cost reduction. In addition, the proposed LED architecture allows for the fabrication of flexible high-brightness LEDs in the blue spectral range provided that an appropriate flexible transparent contact is optimized.

## References

[1] Li S, Waag A (2012). GaN based nanorods for solid state lighting. J Appl Phys.

[2] Shirasaki Y, Supran GJ, Bawendi MG, Bulovic V (2013). Emergence of colloidal quantum-dot light-emitting technologies. Nat Photonics.

[3] Kang MS, Lee CH, Park JB, Yoo H, Yi GC (2012). Gallium nitride nanostructures for light-emitting diode applications. Nano Energy.

[4] Foltynski B, Giesen C, Heuken M (2015). Self-organized growth of catalyst-free GaN nano-and micro-rods on Si (111) substrates by MOCVD. Phys Status Solidi B.

[5] Salomon D, Dussaigne A, Lafossas M, Durand C, Bougerol C, Ferret P, Eymery J (2013). Metal organic vapour-phase epitaxy growth of GaN wires on Si (111) for light-emitting diode applications. Nanoscale Res Lett.

[6] Collet M, Salomon S, Klein NY, Seichepine F, Vieu C, Nicu L, Larrieu G (2014). Large-scale assembly of single nanowires through capillary-assisted dielectrophoresis. Adv Materials.

[7] Lee CH, Kim DR, Zheng X (2011). Fabrication of nanowire electronics on nonconventional substrates by water-assisted transfer printing method. Nano Lett.

[8] Son K, Lee DH, Yi J, Lee WW, Park WI (2011). Synthesis and transfer of Si nanowire arrays embedded in photo-sensitive polymer films for non-planar electronics. J Physics D App Phys.

[9] Liu Z, Xu J, Chen D, Shen G (2015). Flexible electronics based on inorganic nanowires. Chem Soc Rev.

[10] Plass KE, Filler MA, Spurgeon JM, Kayes BM, Maldonado S, Brunschwig BS, Atwater HA, Lewis NS (2009). Flexible polymer-embedded Si wire arrays. Adv Materials.

[11] Spurgeon JM, Boettcher SW, Kelzenberg MD, Brunschwig BS, Atwater HA, Lewis NS (2010). Flexible, polymer-supported, Si wire array photoelectrodes. Adv Materials.

[12] Reimer ME, Bulgarini G, Akopian N, Hocevar M, Bavinck MB, Verheijen MA, Bakkers EPAM, Kouwenhoven LP, Zwiller V (2012). Bright single-photon sources in bottom-up tailored nanowires. Nat Commun.

[13] Anttu N, Abrand A, Asoli D, Heurlin M, Aberg I, Samuelson L, Borgstroem M (2014). Absorption of light in InP nanowire arrays. Nano Res.

[14] Nadarajah A, Word RC, Meiss J, Könenkamp R (2008). Flexible inorganic nanowire light-emitting diode. Nano Lett.

[15] Chung K, Beak H, Tchoe Y, Oh H, Yoo H, Kim M, Yi GC (2014). Growth and characterizations of GaN micro-rods on graphene films for flexible light emitting diodes. Appl Mater.

[16] Park S, Kim H, Vosgueritchian M, Cheon S, Kim H, Hoon Koo J, Kim TR, Lee S, Schwartz G, Chang H, Bao Z (2014). Stretchable energy-harvesting tactile electronic skin capable of differentiating multiple mechanical stimuli modes. Adv Materials.

[17] Park KT, Guo Z, Um HD, Jung JY, Yang JM, Lim SK, Kim YS, Lee JH (2011). Optical properties of Si microwires combined with nanoneedles for flexible thin film photovoltaics. Opt Express.

[18] Koester R, Hwang JS, Durand C, Dang DLS, Eymery J (2010). Self-assembled growth of catalyst-free GaN wires by metal–organic vapour phase epitaxy. Nanotechnology.

[19] Koester R, Hwang JS, Salomon D, Chen X, Bougerol C, Barnes JP, Le Si DD, Rigutti L, Tchernycheva M, Durand C, Eymery J (2011). M-plane core–shell InGaN/GaN multiple-quantum-wells on GaN wires for electroluminescent devices. Nano Lett.

[20] Tchoulfian P, Donatini F, Levy F, Dussaigne A, Ferret P, Pernot J (2014). Direct imaging of p–n junction in core–shell GaN wires. Nano Lett.

[21] PATENT Chen X, Durand C, Eymery J, Salomon D (2012). Process for catalyst-free selective growth on a semiconductor structure. patent number: WO2012136665.

[22] Befahy S, Yunus S, Burguet V, Heine JS, Troosters M, Bertrand P (2008). Stretchable gold tracks on flat polydimethylsiloxane (PDMS) rubber substrate. J of Adhesion.

[23] Sheu JK, Su YK, Chi GC, Koh PL, Jou MJ, Chang CM, Liu CC, Hung WC (1999). High-transparency Ni/Au ohmic contact to p-type GaN. App Phys Lett.

[24] Kalaitzakis FG, Pelekanos NT, Prystawko P, Leszczynski M, Konstantinidis G (2007). Low resistance as-deposited Cr/Au contacts on p-type GaN. App Phys Lett.

[25] Sheu JK, Su YK, Chi GC, Jou MJ, Liu CC, Chang CM (1999). Indium tin oxide ohmic contact to highly doped n-GaN. Solid State Electron.

[26] Wang DF, Shiwei F, Lu C, Motayed A, Jah M, Mohammad SN, Jones KA, Salamanca-Riba L (2001). Low-resistance Ti/Al/Ti/Au multilayer ohmic contact to n-GaN. J Appl Phys.

[27] Jacopin G, De Luna BA, Lavenus P, Rigutti L, Julien FH, Zagonel LF, Kociak M, Durand C, Salomon D, Chen XJ, Eymery J, Tchernycheva M (2012). Single-wire light-emitting diodes based on GaN wires containing both polar and nonpolar InGaN/GaN quantum wells. Appl Phys Express.

[28] Davydov VY, Klochikhin AA, Emtsev VV, Kurdyukov DA, Ivanov SV, Vekshin VA, Bechstedt F, Furthmueller J, Aderhold J, Graul J, Mudryi AV, Harima H, Hashimoto A, Yamamoto A, Haller EE (2002). Bandgap of hexagonal InN and InGaN alloys. Phys Status Solidi B.

[29] Durand C, Bougerol C, Carlin JF, Rossbach G, Godel F, Eymery J, Jouneau P, Mukhtarova A, Butte R, Grandjean N (2014). M-Plane GaN/InAlN multiple quantum wells in core–shell wire structure for UV emission. ACS Photonics.

[30] Hong YJ, Lee CH, Yoon A, Kim M, Seong HK, Chung HJ, Sone C, Park YJ, Yi GC (2011). Visible-color-tunable light-emitting diodes. Adv Mater.

[31] Tchernycheva M, Lavenus P, Zhang H, Babichev AV, Jacopin G, Shahmohammadi M, Julien FH, Ciechonski R, Vescovi G, Kryliouk O (2014). InGaN/GaN core–shell single nanowire light emitting diodes with graphene-based p-contact. Nano Lett.

[32] Wang X, Zhi L, Muellen K (2008). Transparent, conductive graphene electrodes for dye-sensitized solar cells. Nano Lett.

[33] Chang KM, Chu JY, Cheng CC (2004). Highly reliable GaN-based light-emitting diodes formed by p-In0.1Ga0.9N-ITO structure. Photonics Technology Letters. doi: 10.1109/LPT.2004.830523.

[34] Babichev AV, Zhang H, Lavenus P, Julien FH, Egorov AY, Lin YT, Tu LW, Tchernycheva M (2013). GaN nanowire ultraviolet photodetector with a graphene transparent contact. Appl Phys Lett.

